# Cognitive Testing of the People’s Voice Survey Instrument to Assess Adults’ and Adolescents’ Experience in European Healthcare Systems

**DOI:** 10.3390/healthcare14111566

**Published:** 2026-06-03

**Authors:** Katrīne Kūkoja, Inese Stars, Ģirts Briģis, Mariana Lopes Simões, Emma Clarke-Deelder, Anita Villeruša

**Affiliations:** 1 Institute of Public Health, Rīga Stradiņš University, LV-1007 Rīga, Latvia; inese.stars@rsu.lv (I.S.); girts.birigis@rsu.lv (Ģ.B.); anita.villerusa@rsu.lv (A.V.); 2 Vidzeme University of Applied Sciences Scientific Institute, LV-4201 Valmiera, Latvia; 3 Friede Springer Endowed Professorship for Global Child Health, Witten/Herdecke University, 58448 Witten, Germany; mariana.lopes@uni-bielefeld.de; 4 Graduate School “Health Policy and Systems in Uncertainties” (GRASP)/AG3: Epidemiology & International Public Health, Faculty of Public Health, Bielefeld University, 33501 Bielefeld, Germany; 5 Swiss Tropical & Public Health Institute, 4123 Allschwil, Switzerland; emma.clarke-deelder@swisstph.ch; 6 University of Basel, 4051 Basel, Switzerland

**Keywords:** People’s Voice Survey instrument, cognitive interviews, Tourangeau’s 4-step model, text summary approach, healthcare system, adolescent, adults, survey adaptation

## Abstract

**Background/Objectives**: Understanding people’s experiences is essential for improving healthcare systems (HCSs) and health outcomes, but assessing these experiences requires accurate, culturally and contextually appropriate measures. The People’s Voice Survey (PVS) is an instrument developed to assess current and future user experience across all levels of HCS globally. This research presents a cognitive interview (CI) approach as one measure for adapting PVS questions for adult and adolescent populations in three European countries. **Methods**: Twenty-four semi-structured CIs were conducted in Latvia, fifteen in Switzerland, and four in Germany. Study participants were recruited via convenience sampling. Interviews were conducted across virtual, telephone, and face-to-face settings. The “text summary” approach and Tourangeau’s four-step question-and-answer model served as the theoretical framework for the study. **Results**: The majority of the core PVS questions tested functioned well. The most common issues identified were related to comprehension and the decision process in all country cases. Analysis showed that almost all PVS core questions were also suitable for adolescents, with the exception of some questions related to evaluation and knowledge of HCS functioning, due to limited knowledge and/or experience with HCSs. Key adjustments included clarifying the question formulation, adding explanations to unclear and unknown terms and concepts, adding the following questions and missing answer categories. **Conclusions**: The paper shows how CIs are used to identify issues at all response stages when answering PVS core questions, thereby enabling the measure to be improved and supporting the use of CIs to enhance HCS assessment instruments.

## 1. Introduction

In 2022, 1.1 million deaths among people under the age of 75 in the European Union were considered avoidable, including 386,710 treatable deaths (89.7 per 100,000), which could have been prevented with early treatment or disease prevention [1]. This shows the need to improve the access to and quality of health care systems (HCSs). However, reaching these goals requires shifting the focus of HCS design from disease and health institutions to people, emphasising equity in access, quality, responsiveness, participation, efficiency, and HCS resilience. HCSs must serve and engage with the people they are designed to serve, taking their lived experiences, preferences, and social context into account, especially in the case of vulnerable groups. Therefore, understanding how HCSs perform is essential, and assessing user experience is one way to do so [2,3,4].

The PVS is one of the few cross-nationally comparable instruments for tracking population-level perspectives on HCS performance developed by the Quality Evidence for Health System Transformation (QuEST) Network. The first wave of surveys (2022–2025) included 19 countries, and by the second wave, the PVS had already been conducted in 25 countries [5]. Unlike traditional patient experience surveys that focus mainly on recent service users, the PVS captures perspectives from a representative sample of the overall adult population, including past, current, and potential future users. The survey has mainly been implemented using telephone-based and online approaches, with supplementation of in-person surveys in areas with low phone coverage to ensure nationally representative information. Data from the PVS can be used to give policymakers insights into how well national HCSs meet the needs of all groups, beyond specific age categories or disease cohorts, supporting the development of more effective, people-centred HCSs while enhancing accountability to populations [5,6,7].

Until recently, the PVS was used exclusively with adults (18+ years), as it was originally developed for adult respondents. However, there is increasing interest in also using it to understand the experiences and perspectives of adolescents. Recent and ongoing data collection in Latvia, Switzerland, and Germany also includes adolescent participants under age 18, motivated by the importance of adolescence as a unique and critical stage of human development that establishes the foundations for good health [8]. It is a time to adopt behaviours that are responsible for the vast majority of premature mortality, connected to nutrition, physical activity, and substance use. Despite the general notion that adolescence is a healthy period, increasing evidence suggests rising disease and mortality burdens in this age group. For the first time in over 20 years, an increase in the median 15–19-year mortality rate has been observed in the WHO European Region, with increases reported in more than a third of countries (19) across the Region [9]. Many countries worldwide have also seen significant increases in the burden of mental illness in this age group [10]. Given the above, it is essential to understand the extent to which the HCSs meet the needs of this target group. However, adolescents are often excluded, both from health services and research, including surveys, due to the need for consent from parents or guardians [11].

To ensure that the results from the PVS are valid and comparable, it is critical to ensure that the survey questions are well understood in this population and interpreted the same way among both adults and adolescents. Cognitive interviews (CIs) are among the core methods used in adapting survey instruments to a national context, providing insights into the participants’ understanding of, as well as ability and willingness to answer, survey questions, thereby ensuring that respondents interpret questions as intended and provide accurate responses, reducing measurement error, and improving the face validity of quantitative surveys [12,13,14]. For survey instruments intended for use in multiple languages, CIs can help identify whether issues stem from translation errors, context-specific interpretation differences, or inherent design flaws in the original instrument. Such insights enable targeted revisions to survey instruments before use [15,16,17]. During the development of the PVS (first wave of interviews), QuEST researchers had already conducted over 80 CIs in eleven countries and seven languages to establish the instrument’s face validity. They highlighted multiple areas for improvement, including reducing item length and simplifying high-cognitive-burden questions [5]. As part of the survey adaptation process in Latvia, Switzerland, and Germany, additional CIs were conducted with both adolescents and adults using the amended PVS instrument.

This study aimed to assess how respondents understand and interpret the PVS questions, identify and eliminate potential ambiguities, and improve the quality of survey data gathered in Latvia, Switzerland, and Germany by ensuring the cultural appropriateness of the survey instrument.

This paper outlines the process of reviewing and revising the PVS core questions and presents insights from CIs, examining the cognitive processes involved in completing surveys in three European country cases. It also shows how CIs can be incorporated into questionnaire adaptation to enhance data quality and reduce errors across four stages of answering survey questions [18].

## 2. Materials and Methods

### 2.1. Study Design and Survey Instrument

This study used a qualitative research approach to adapt the original PVS instrument for use in Latvia, Germany, and Switzerland. The core instrument consists of 52 questions structured in the following sub-chapters: 1.1. Basic demographics; 1.2. Health Status; 1.3. Patient activation; 2.1. Usual source of care; 2.2. Utilisation; 2.3. System competence in population health; 2.4. Non-use of care; 3.1. Care competence and user experience; 3.2. Facility endorsement; 4.1. Assessment of the public health system; 4.2. Overall health system assessment; 4.3. Expectations for health system quality and final questions. CIs were used to assess instrument performance and inform final revisions [5].

Before conducting CIs, several steps had already been taken, and approaches differed slightly among countries. In Latvia and Switzerland, the survey instrument was first translated from English into the national languages (Latvian in Latvia; French, Italian, and German in Switzerland) and then back-translated into English for quality control, following recommendations from the PVS developers [5]. The second step involved interviews with experts, who provided feedback on the suitability of the PVS instrument for Latvian and Swiss HCSs, with a focus on terminology and organisational aspects. CIs were then conducted using the revised translated instrument in each country. The adaptation process in Switzerland and Latvia was completed through pilot testing. During each stage, the survey instrument was refined in each country.

In Germany, the instrument also underwent cross-cultural adaptation. The original English version was first adjusted to better reflect the German cultural and linguistic context. Two independent German translations were then produced: one by the university research team and another by a certified translator. The translations were compared and reconciled through a qualitative review involving both translators and an independent mediator. Finally, CIs were conducted with youth participants, resulting in final refinements to the German questionnaire prior to implementation.

This paper examines the CI process and findings for each country, focusing specifically on the core PVS core questions cognitively tested in at least two countries (*n* = 31). Additional questions were tested in each country; these are excluded from the present analysis to ensure comparability across countries. 

### 2.2. Setting and Participants

Within the framework of the project “Adolescent and Adult Perspectives on European Health System Performance: A Four-Country Study”, the study was conducted by the Institute of Public Health at Rīga Stradiņš University in Latvia and the Swiss Tropical and Public Health Institute in Switzerland. Although Germany was not formally part of this project and independently adapted the instrument for use with an adolescent population, the research team considered it valuable to include the German results in the study to provide broader insights into the instrument’s adaptation across different country contexts and age groups, including adolescents. The Friede Springer Endowed Professorship for Global Child Health at Witten/Herdecke University conducted the study in Germany.

In Switzerland and Latvia, a convenience sampling approach was employed. When selecting study participants, it was important to ensure heterogeneity in characteristics that may affect how the questions are understood, reflecting the expected PVS population, maintaining a balance across gender, age, education, and, in the case of Latvia, place of residence (capital city, other city, or rural area).

Participants were recruited using “network-based” sampling combined with the snowball technique in both Switzerland and Latvia. In Latvia, the inclusion criterion for study participants was that they speak Latvian as their native language. In Switzerland, separate samples were selected for German-, French-, and Italian-speaking participants.

In Germany, panel-based convenience sampling via a market research company was used. Within this pool, participants were purposively selected according to predefined criteria. Additionally, for Germany, eligibility required that none of the participants had taken part in a qualitative study within the past six months. Selection criteria aimed to achieve a balanced mix in terms of gender, age (15–18 years), and educational background. In addition, recruitment was conducted nationwide to ensure a broad regional distribution. All participants met the technical requirements necessary for taking part in an online interview.

### 2.3. Data Collection

Each country employed a slightly different approach for conducting CIs. In Latvia, CIs with native speakers (Latvian language) were conducted in two rounds: first with the adult population (June 2025) and, after improving the instrument based on the first round’s feedback, a second round with adolescents (15–19 years of age, July to September 2025) by three interviewers. In Switzerland, CIs with native speakers (French, German, and Italian) were conducted in a single round, simultaneously among adolescents and adults, from February to March 2025, by two interviewers (one native French speaker and one native bilingual French-German speaker). Additional CIs were conducted in the languages spoken by migrant groups targeted for participation in the Swiss and Latvian surveys, but these were not included in the present analysis, which focuses only on interviews in national languages. In Germany, the PVS survey included a sample of adolescents aged 15–18 to facilitate robust subgroup analysis. This approach ensured that the instrument was assessed within the survey’s primary target population, allowing the identification of age-specific issues that might not emerge in a broader sample. Interviews in Germany were conducted by a single interviewer in November 2024.

Members conducting interviews in each country were trained and experienced in CI techniques. To enhance the unified approach to conducting CIs, the Swiss Tropical and Public Health Institute shared training materials on CI approaches with the Latvian and German teams. Researchers conducted semi-structured CIs using either a concurrent or a retrospective approach, employing the think-aloud method, where respondents were asked to verbalise their thoughts in real time while or after answering questions, with probing questions as needed, such as when a participant appeared confused, contemplative, uncomfortable, or otherwise showed a noticeable ‘reaction’ to a question. Probes were designed during interviews based on emerging problems and/or developed in advance [17].

The interview approach was grounded in Tourangeau’s (1984) [18] four-step survey response model, which outlines the stages respondents undergo when answering questions—comprehension, retrieval, decision, and response. These four steps can occur sequentially or in parallel, depending on the respondent and the question asked. Table 1 presents examples of CI probes, organised by the Tourangeau four-step model [17,18].

In Latvia and Switzerland, CIs were conducted using a virtual online video platform (Zoom, MS Teams), telephone, and face-to-face interviews, while in Germany, all four CIs were conducted virtually. The mode of CIs was aligned with the survey administration methods used in each country. All CIs lasted approximately 60 to 90 min. Respondents provided brief descriptive information before the interviews to obtain sociodemographic data from participants.

### 2.4. Data Analysis

Qualitative analysis was conducted using the “text summary” approach defined by Willis (2015) [19]. This is a commonly used approach to summarising CI results, focusing on identifying dominant themes, conclusions, and problems arising across all four stages of the question-answering process [18,19]. Text summaries in each country were developed at two levels: (1) an interview-level text summary, where, after each interview, the interviewer recorded structured notes into shared analysis documents, representing summaries of each CI per question tested; and (2) a question-level text summary, which summarised key findings, identifying patterns and themes among all CIs in each PVS core question. Please see Table A2.

**Figure 1:**
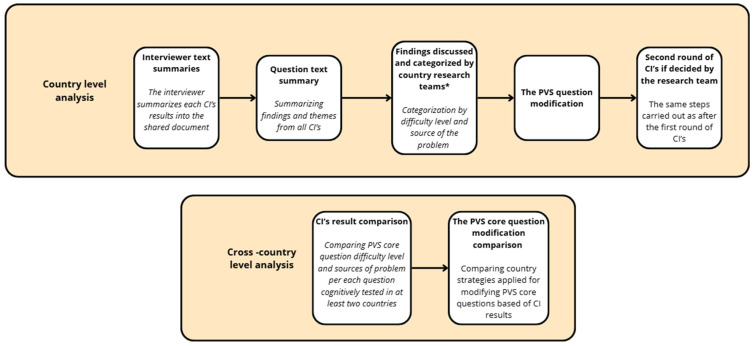
Process of CI data analysis in Latvia, Switzerland, and Germany [18,19]. * Members of the Latvian research team oversaw the coding of answering-stage problems and sub-problems in all three countries.

After each round of interviews, feedback was discussed among country-level research team representatives, with diverse qualifications and backgrounds, and the PVS questions were coded into the following categories: (a) never challenging, (b) rarely challenging (the question was found to be challenging for up to two respondents per target group in the case of Latvia and Switzerland, and for one respondent in the case of Germany), and (c) frequently challenging (the question was challenging for more than two respondents per target group in the case of Latvia and Switzerland, and for more than one respondent in the case of Germany). Frequently and rarely challenging questions were also categorised by problem source using Tourangeau’s 4-Step Model based on the question text summary. This analysis method has also been applied in other studies, where CIs have been used to adapt survey instruments [12,20]. To ensure consistency across the multi-country research teams, Latvian research team members oversaw the coding of answering stage problems and sub-problems in all three country cases. Figure 1 illustrates the steps undertaken for data analysis.

Based on the conclusions after each round of CIs, the PVS questionnaire was modified, in the majority of cases, for rarely and frequently challenging questions. The PVS questions were either improved, the target groups were altered, or the questions remained unchanged. Additionally, after analysing data at the country level, the CIs’ results were compared across countries for the identification of common themes, while taking into consideration cross-country comparison limitations due to methodological differences. When reporting the results of the study, the Cognitive Interviewing Reporting Framework was taken into consideration [21].

## 3. Results

### 3.1. Participant Characteristics

A total of 24 individuals in Latvia participated in the CIs. Of those, eight were adolescents (ages 15–19) and 16 were adults (ages 20+). The majority of respondents were female(16 respondents; eight males), and the interviewees had diverse educational levels: nine respondents had a level of education below secondary, six had secondary or vocational secondary education, and nine had higher education. Of the respondents, 13 lived in the capital city of Latvia, seven in regional cities, and four in rural areas.

In total, 15 individuals in Switzerland participated in CIs, of whom four were adolescents (15–19) and 11 were adults (ages 20+), including 11 female participants and four male participants. Education and place of residence were not recorded for the Swiss participants, but interviewers were instructed to recruit participants with diverse educational backgrounds.

In Germany, the sample comprised four adolescents aged 15–18 years, two female and two male. Three respondents lived in capital cities, and one lived in a rural area. At the time of the interview, all participants had an educational level below secondary.

### 3.2. Overall Results

In total, 47 core PVS questions were tested with both adults and adolescents in Latvia. Overall, the first CI round among adults identified 28 rarely challenging PVS core questions and seven frequently challenging questions, indicating that 74.5% of the questions tested had some level of difficulty. The second round of testing among adolescents, following the first modifications of the survey instrument, identified 13 questions as rarely challenging and nine as frequently challenging, reducing the frequency to 46.8% in Latvia. 3 out of these twenty-two somewhat problematic questions were challenging only in the second round.

Of 21 core PVS questions tested in Germany, 12 were coded as rarely challenging and seven as frequently challenging (90.5%), whereas in Switzerland, 11 were rarely challenging and four were frequently challenging (65.2%). Please see Table 2.

**Figure A1:**
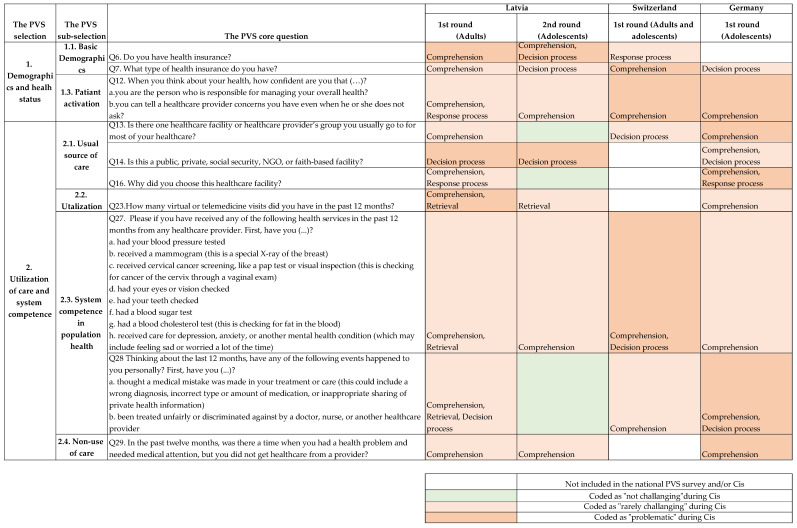

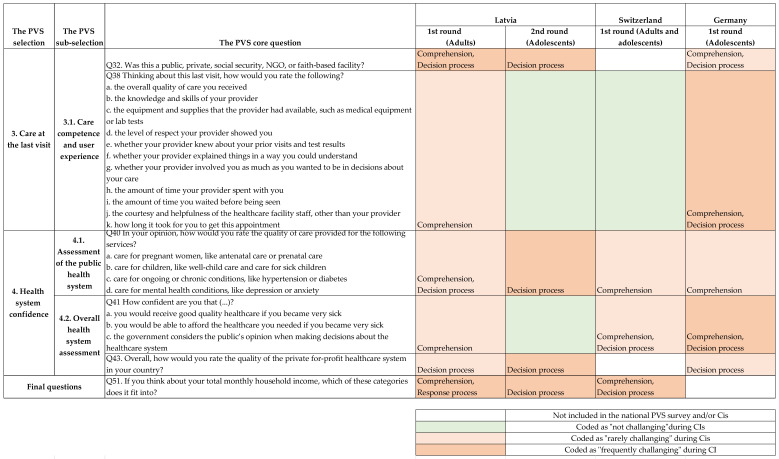
CIs results structured by country and Tourangeau’s 4-Step Model.

Analyses assessing common patterns across countries showed that, of the 31 PVS questions tested in two countries (*n* = 18) or all three countries (*n* = 13), 16 were coded as frequently challenging in at least one country. Of these 16 questions, four had the same dominant difficulty type, mainly related to comprehension (see Figure A1). The majority of these questions were challenging due to comprehension issues (see Table 3), and the second most frequent problem concerned the decision process.

In most cases, identified problems were addressed when detected in two or more CIs (in the case of Germany, when detected in one or more CIs, due to the limited sample size). 13 questions in Latvia during the first round of CIs, and 11 questions during the second round (8 questions for the second time) were modified, 6 questions in Switzerland, and fourteen questions in Germany. Refer to Table 4 and Table 5 for examples of question modifications.

Comprehension issues in all three countries were mainly addressed by refining the question-and-answer formulation and providing explanations for unclear or unknown words in the questions. Meanwhile, retrieval issues were primarily addressed by providing response categories for those unable to provide exact numbers, as suggested during CIs, to reduce the recall burden. Response process issues were primarily addressed by adding missing answer categories or adjusting existing ones in the survey instrument.

When addressing decision process issues, which were more commonly observed among adolescents, countries adopted different approaches. In Latvia, the tendency was to omit the question from the adolescent survey, whereas in Switzerland and Germany, there was a tendency to include an answer category for “Don’t know”. For instance, due to a lack of knowledge and/or experience in HCSs, questions about household monthly income, healthcare insurance, type of medical facility, evaluation of the quality of various healthcare services, and overall healthcare quality in Latvia were removed from the adolescents’ PVS survey in Latvia.

### 3.3. Comprehension

While other answer process stage difficulties were concentrated in specific PVS sub- sections, comprehension problems were observed across all sub-sections, indicating challenges in how well respondents interpret the intent behind the question. Comprehension difficulties were present in 15 of 16 analysed questions (13 of which were frequently challenging in at least one country), and in 10 questions, comprehension issues overlapped across countries. For more detailed information, please see Table A2.

Although differences were observed among countries in comprehension problems, the problem types were consistent and were linked with three problem codes in all country cases: (1) unknown, unclear, or complex terms and concepts; (2) ambiguity (different or unclear interpretation); (3) question length and structure complexity. Please see Table A1.

Comprehension problems related to unfamiliar, unclear, or complex terms and concepts were the most common in all three country cases. Insurance- and HCS-related terms, such as “private health insurance”, “insurance policy”, as well as insurance models in the Swiss case, were repeatedly challenging (Q6, Q7), since formal and administrative labels didn’t align with cognitive response models. Also, preventive and diagnostic service terminology, like “mammography” and “pap-test”, was not understood as intended in all cases (Q27). For example, the most challenging question in both target groups in Latvia was “Q6 Do you have a private health insurance?” because the term “private” confused respondents, who were uncertain whether to include employer-provided insurance in their response.

In addition to HCS-specific terminology, the terms “household” and “household total after-tax income” (Q51) were identified as frequently challenging in both Latvia and Switzerland (not tested in German CIs). Here, respondents applied divergent interpretations, expressed uncertainty about shared residence versus shared budget, and about the inclusion of benefits and the income of other household members.

Some PVS questions were also challenging due to ambiguity: respondents understood the words in the question, but the intended meaning or reference frame was unclear, leading to differences in interpretation. For example, phrases such as “quality of HCS” (Q38), “afford quality care” (Q41), “very sick” (Q41), and “needs of your age group” (Q41) were identified as subjective and elicited different personal interpretations, leading to inconsistent responses. Some of the questions were perceived as even contradictory. For example, the term “medical facility or practice”, included in multiple questions (Q13, Q32), was difficult for respondents in Latvia because they did not know whether they were expected to consider healthcare institutions, practices within institutions, or individual providers when answering. After receiving feedback from adults, the wording of these questions was revised, improving comprehension among adolescents.

Several questions were also perceived as too detailed, long, and/or complicated (Q16, Q27, Q38, Q12, Q23), especially in the telephone interview context, where respondents reported that questions were more difficult to process, remember, and answer, increasing their cognitive overload. Please see Table A1.

### 3.4. Retrieval

Three out of 16 PVS core questions analysed showed retrieval issues (Q23, Q27, Q28; one frequently challenging, two rarely challenging), indicating difficulties retrieving past experiences to answer accurately. These questions were challenging only in the Latvian sample, exclusively concentrated in the PVS selection “Utilisation of care and system competence”, and stemmed from a 12-month recall period. These questions were even more challenging for those who use healthcare services more frequently, as well as for parents and caregivers, who found it difficult to separate personal from proxy experiences. Consequently, adults had more retrieval problems (three questions) than adolescents (one question). For example, question Q23, “How many virtual or telemedicine visits did you have in the past 12 months?”, was coded as frequently challenging among adults and rarely challenging among adolescents. Most CI respondents emphasised that the recall period was too long to accurately remember and count all calls or text consultations over the past 12 months and suggested shortening it to at least six months or providing response ranges instead of requiring precise counts.

### 3.5. Decision Process

Issues with the decision process, linked to difficulties in interpreting the question in a way to make a decision about how to answer, were the second most common problems encountered across all country cases. Overall, 12 out of 16 questions were rarely challenging (*n* = 2) or frequently challenging (*n* = 10) in at least one country. These problems were most frequently observed in questions about confidence in and evaluation of HCSs, as well as utilisation of care and system competence. Although the same PVS selections typically generate decision process problems across all countries, differences were observed. Decision process issues overlapped across four questions among all country cases, whereas the situation differed across eight questions. Please see Table A2 and Figure A1.

Decision process problems stemmed from two main reasons: either a lack of experience or knowledge of HCSs, or a reluctance to respond due to the topic’s sensitivity.

When it comes to a lack of knowledge and/or experience in HCSs, questions about how HCS functions were frequently challenging for both adolescents and adults. For example, identifying the ownership type of healthcare facility (Q14, Q32) was considered frequently challenging in Latvia and Germany (not included in the Switzerland CIs). Nearly all respondents stated they would not know the answer to this question, even if an explanation were given.

Decision process problems appeared more frequently and strongly among adolescents, even in questions about basic demographics, for example, questions about having health insurance (Q6, Q7), where adolescents in Latvia and Germany did not know the answer to the question, stating that, in most cases, healthcare is still managed by their parents. Besides questions about HCS organisation and evaluation, adolescents in the Latvian and Swiss samples also found questions about the estimated total monthly household net income frequently challenging (Q51). While they understood the question, they did not know the answer.

Regarding topic sensitivity, only one question in the Swiss case—Q27 (option k), which asked whether the respondent had ever received care for depression, anxiety, or another mental health condition within the past 12 months, was considered slightly sensitive, as one respondent indicated that they would prefer not to answer.

### 3.6. Response Process

Of the 16 analysed PVS core questions, four presented response issues (one overlapping across country cases), indicating difficulties in mapping responses or aligning them with the provided response options. Difficulties occurred in the following PVS sub-sections: Basic demographics (Q6); Patient activation (Q12); Usual source of care (Q6); and one question in the final section of the survey (Q51).

The main problems with the response process stemmed from an incomplete list of response categories and, less frequently, from the need to select multiple answers rather than a single one. However, only one question was identified as frequently challenging in the response process in Latvia: Q51, which asked about measuring household income, because higher-income answer categories were needed. Also, in Germany, one question (Q16) about the main reason a person chose a specific healthcare facility or medical practice for primary care was identified as frequently challenging because of the difficulty of selecting just one answer from the provided options.

## 4. Discussion

This paper describes a process for collecting feedback on the PVS core questions in the Latvian, German, and Swiss PVS instruments, which were identified as frequently challenging in at least one of the countries during CIs. As in other studies [13,14,20,22], CIs helped identify common pitfalls in survey design and measurement instruments.

CIs have been used to improve survey instruments for healthcare assessment, with a focus on patient-reported outcome measures [23]. However, a limited number of studies describe the use of CIs to develop and adapt holistic, user-centric instruments assessing HCS performance, and, if they do, they focus mainly on instruments covering specific aspects of HCSs. One example is Richmond et al.’s (2022) study, in which CIs were used as one of the instruments to validate a newly developed survey on trust in healthcare specialists [24].

CIs have also been used in previous studies to pretest survey questions with people younger than 18 [14,25,26,27], supporting their use for adapting survey instruments for younger populations. Extending CIs to this age group is highly valuable, as youth offer a unique perspective that helps identify problems adult respondents may not recognise and helps develop instruments suitable also for the adolescent population. LaPietra et al. (2020) [14] conducted a study in which CIs were used to test a custom-developed youth survey designed to evaluate a youth program. CIs were conducted with 10 respondents, aged 10 to 13 years and 15 to 17 years. The study found that even within these age groups, there were differences in understanding survey items, with younger respondents having more comprehension problems [14]. This further underscores the importance of assessing different age groups during cognitive testing, as they differ in cognitive development. Additionally, including adolescents in HCS assessment surveys is essential to further improve this age group’s health outcomes, their ability to participate in health-related decision-making, their access to confidential medical counselling and to health services suitable for their maturity, in accordance with The UN Convention on the Rights of the Child [28].

Consequently, this study contributes to a small but growing literature on the use of CIs to improve survey instruments that capture people’s experiences and perceptions of HCSs holistically, supporting the development of a valid and reliable survey measure aligned with Tourangeau’s 4-Step Model [18]. It is the first study to focus specifically on the PVS in both adult and adolescent populations in three European countries.

Our study findings suggest that respondents sampled from the Latvian, German, and Swiss general populations are likely to answer the majority of PVS core questions without major issues or any need for major question modifications. Analysis showed that many questions posed minor challenges to many participants, so they were counted as “frequently challenging” even though they were easy to address. Findings in Latvia are consistent with prior research on CI analysis based on Tourangeau’s (1984) 4-Step Model, showing that respondents encounter problems at each step of the answering process when responding to survey questions [12,18,20].

Similar to other studies [14,20,29], the most common problems in the answering process were related to comprehension, arising mainly from the translation process.Additionally, for most respondents in Latvia, a 12-month retrieval period was reported as cognitively demanding, especially among those with children or who used health care services extensively. Consequently, retrieval problems were more common among adults, since they usually have more complex and frequent healthcare use, which increases the recall burden. These kinds of patterns have also been observed in other studies. For example, a study conducted in India, in which 21 mothers participated in CIs to enhance survey questions on maternal and child nutrition interventions, found that even a 6-month period was frequently challenging and created temporal confusion [29].

All three country cases demonstrated that many respondents had incomplete knowledge and understanding of the HCS organisation and its functioning, which impeded their ability to answer questions on these topics. This may be because national HCSs are overly complicated or lack transparency, because the public is not sufficiently informed about them, or because when the public is sufficiently informed, the information is not presented in easily understandable language. Consequently, this also affects how effectively people can navigate the system [30].

Additionally, preliminary age-specific results show that while questions about HCS functioning—including its organisation and funding schemes—were difficult for both adolescents and adults, questions about HCS evaluation were more challenging for adolescents. These differences could partly be explained by differences in HCS experience and knowledge, as well as by the fact that, in most cases, healthcare is still organised and mediated by their parents or caregivers, indicating an age-related decision burden, especially among younger adolescents (15–17 years of age). This leads to uncertainty about responsibility, eligibility, and what could be considered “their experience”, thereby complicating the decision process [31]. Further research should be conducted to explore these tendencies.

There were differences among country cases with regard to question difficulty levels and the answer stage, mainly for questions that produce retrieval, decision, and response process difficulties. These differences could stem from the translation (language differences) and general differences in the functioning of national HCSs, including the roles of public and private sectors, appointment systems, etc., as well as country-specific HCS terminologies and cross–cultural differences, for example, in answering sensitive questions about discrimination and mental health. Similar tendencies were observed by Miller et al. (2011) [32], who developed an evidence-based methodology to assess the comparability of survey questions across cross-cultural contexts. Study results of 135 CIs reported interpretive patterns due to sociocultural and language-related differences among countries [32].

When interpreting the results of this study, it is important to consider the study’s limitations, the most important of which is the lack of a unified approach to conducting CIs across countries, affecting both cross-country and age-specific comparisons. For example, not all countries tested survey questions in both populations. In Germany, CIs were conducted only with adolescents; in Latvia and Switzerland, interviews were conducted with both adolescents and adults. Furthermore, in Latvia, the survey instrument was modified after the first round of CIs, so a comparison between adult and adolescent CIs in Latvia was not fully possible. These inconsistencies introduce significant confounding variables, making direct comparisons problematic and affecting the validity of their cross-country synthesis. Further studies aiming to assess cross-country comparisons should follow a more harmonised protocol in conducting and analysing CIs.

This study also presents strengths, including recruiting CI respondents from diverse sources to ensure as much similarity to the target population as possible. However, as with any pre-testing, it should not be assumed that responses from main survey participants will be identical, especially in Germany, where only four CIs were conducted. As a result, the German findings are more preliminary, which decreases direct comparability with the Latvian and Swiss cases. Nonetheless, small sample sizes are common in CIs [14,17,27,33].

Another study strength was that Latvia tested the instrument in two rounds. As a result of the first modifications to the instrument, the number of respondents who, due to comprehension and answer-process challenges, were unable to answer the question was reduced. Consequently, the second round of testing showed significantly fewer comprehension, retrieval, and response-process issues. However, in Germany and Switzerland, only one round of testing was conducted, which is not uncommon for CIs [12,17,29,34]. In all three countries the CIs were intended to supplement a pilot study that was carried out in each country afterwards, not to replace it, and helped improve the survey instrument beforehand.

Research findings demonstrate that CIs are an effective method for survey adaptation, identifying problems that would otherwise go undetected. A study showed that even small changes substantially improve respondents’ answers and reduce non-response and response errors. However, when adapting the PVS to specific HCSs and populations, researchers recognised that, in some cases, potential improvements may reduce comparability with prior PVS data-collection rounds. Therefore, although CI findings were and should be considered to a great extent, the final decision by country research teams also takes into account other relevant factors.

## 5. Conclusions

Conducting CIs with cross-sectional samples of the general population helped develop country-level PVS instruments tailored to the intended audiences in Latvia, Switzerland, and Germany. CI results showed that difficulties in the majority of cases in all three countries stemmed from specific languages, cultures, and respondents’ knowledge and age, but there were also issues more related to the topic of the question and the conceptual success of the question formulation—overly long and complicated questions, questions with long retrieval periods, and detailed questions about HCS functioning.

This study calls for the use of CIs when adapting the survey instrument, considering both the complexity of national HCSs and the target groups’ ability and willingness to respond to survey questions. Although the findings are specific to the PVS, they may be useful for adapting similar survey instruments with adults and adolescents.

## Figures and Tables

**Table 1:** Examples of probing questions per Tourangeau’s four-step model.

Purpose of Interview Questions	Example of “Probing” CI Questions
Comprehension—interpreting the question’s wording and intent.	*Could you explain, in your own words, what this question is asking?*
Retrieval—retrieval of the information needed to answer the question.	*Was it easy or difficult to recall the information you needed to answer this question? Why?*
Decision process—evaluating the recalled information and deciding how to formulate a response.	*Did you hesitate before choosing your answer? Why?*
Response processes—mapping the inner answer to the question onto the survey’s response options.	*Did the response options align well with your internal answer?*

**Table 2:** The PVS core question difficulty level assessment by target groups and countries.

Question Difficulty Level	Latvia		Switzerland	Germany
	1st Round (Adults, *n* = 16)	2nd Round (Adolescents, *n* = 8)	1st Round (Adults and Adolescents, *n* = 15)	1st Round (Adolescents, *n*= 4)
Frequently challenging	7	9	4	7
Rarely challenging	28	13	11	12
Never challenging	12	25	8	2
Total number of questions tested	47	47	23	21

**Table 3:** Frequently challenging PVS core questions * (*n* = 16) categorised in accordance with Tourangeau’s 4-Step Model.

Response Steps	Latvia		Switzerland	Germany
	1st Round (Adults)	2nd Round (Adolescents)	1st Round (Adults and Adolescents)	1st Round (Adolescents)
Comprehension	14	4	7	12
Retrieval	3	1	0	0
Decision process	4	7	4	7
Response process	3	0	1	1
Questions with difficulties in more than one answer process step.	8	1	3	6

* Table includes 16 questions, which were coded as frequently challenging in at least one country.

**Table 4:** Modifications made to PVS core questions * after CIs by country and age group **.

Modification Type	Latvia		Switzerland	Germany
	1st Round (Adults)	2nd Round (Adolescents)	1st Round (Adults and Adolescents)	1st Round (Adolescents)
Question formulation improved	10	4	2	9
Answer category formulation improved	3		2	4
Additional answer categories added	1	1	1	6
Question removed		7	1	
Following question added	1			
Number of changes made	3	5	4	0
Number of questions tested	16	16	10	14

* The analysis included 16 questions, which were coded as frequently challenging in at least one country. ** In some cases, multiple question modifications were carried out.

**Table 5:** Examples of PVS core question modifications in Latvia, Switzerland, and Germany.

Country	Original Question	Modified Question	
Latvia	Q6. Do you have a private health insurance? 1 Yes 2 No	**After 1st round** Q6. Do you have an additional health insurance? 1 Yes 2 No	**After 2nd round** Q6. Do you have a purchased private health insurance? 1 Yes 2 No
Switzerland	Q11. Which compulsory health insurance model do you have? 1 Standard model (free choice) 2 HMO model (a network of affiliated health care providers) 3 Telemedicine model 4 Family Doctor model 5 Mixed model 6 Other (specify)	Q11. In Switzerland, you have the option of different insurance model that determine who you contact first in case you need healthcare. Which of the following models do you have? 1 Standard model (You can decide for yourself who will treat you. You can go directly to specialists.) 2 HMO model (The first point of contact is your chosen doctor’s group practice or health centre. The practice or health centre then plans and coordinates the further course of treatment) 3 Telemedicine model (The first point of contact is the helpline. Medical professionals will advise you on the next treatment steps over the phone.) 4 Family Doctor model (The first point of contact is your chosen family doctor.) 5 Other (specify)	
Germany	Q13 Is there a healthcare facility or practice (including psychotherapy or alternative medical methods, such as naturopaths) that you usually visit for the majority of your healthcare? Please do not include pharmacies and/or drugstores. 0 No 1 Yes	Q13. Is there a practice or other healthcare facility that you usually visit for the majority of your healthcare? (Psychotherapy or alternative medical methods, such as naturopaths, are included. Pharmacies and/or drugstores do not count). 0 No 1 Yes	

## Data Availability

The data analysed in the current study are not publicly available to protect the privacy of the interviewees. However, they are available from the corresponding author upon reasonable request.

## Abbreviations

The following abbreviations are used in this manuscript:

CI	Cognitive interview
HCS	Healthcare system
PVS	People’s Voice Survey
QuEST	Quality Evidence for Health System Transformation Network

## Appendix A

### Appendix A.1

**Table A1:** Overview of comprehension issues identified in cognitive interviews by country and target group.

Problem Code	Latvia		Germany	Switzerland
	Adults (1st Round)	Adolescents (2nd Round)	Adolescents (1st Round)	Adolescents and Adults (1st Round)
Unknown, unclear, and complex terms and concepts	“private health insurance” (Q6; Q7) “healthcare provider” (Q27) “an unauthorized leak of your personal health data” (Q28) Q32 answer option No 3—“mixed property” “household”, “household income” (Q51)	“insurance policy” (Q6) “mammography”, “faecal tests”(Q27)	“insurance policy” (Q6) “Medizinische Fachperson” (Q12) “Gesundheitsleistungen” (Q14) “medical facility or practice” (Q13) “Pap-Test” (Q27) “Erkankungen des Bewegungsapparats”; “Darmspiegelung” (Q40)	“Standard model (free choice)”; “HMO model (a network of affiliated health care providers)”; “Telemedicine model”; “Family Doctor model”; “Mixed model” (Q7) “total net annual household income” (Q51)
Ambiguity (different or unclear interpretation);	Q12_a “medical facility or practice” (Q13; Q32) Q23 Q29 Q38 answer options g, h, and i “quality of the following healthcare services” (Q40) Q41_b—“afford qualitative healthcare”	Q12_a (Q29)	Q12 a “virtuelle oder telemedizinische Besuche” (Q23) Q28 c—“während der Gesundheitsleistung” Q29—severity of the “gesundheitliches Problem” Q32 Q38 a; b; d; e; g; i; j;k Q40 a; b; d; i Q41 a; b	Q27 „very sick” (Q41_a) “the needs of your age group” (Q41_d)
Question length and structure complexity	Q16 Q27 Q38 Q40		Q12 Q13 Q23 Q40	Q45

### Appendix A.3

**Table A2:** Overview of comprehension issues identified during CIs, presented by country and target group.

Question Tested	Text Summary of Issues Identified	Difficulty Level
Q12. When you think about your health, how confident are you that you are the person who is responsible for managing your overall health? Very confident Somewhat confident Not too confident Not at all confident	Some participants were not sure how to understand the use of ‘confident’ here. Is this about confidence in their ability to manage their own health, or certainty that they are the ones responsible? Other participants focused on the idea of lifestyle (whether they are able to live a healthy lifestyle). They just noted different uses of the term in different questions.	Frequently challenging
